# Physics-Inspired Single-Particle Tracking Accelerated with Parallelism

**DOI:** 10.1101/2025.05.30.657103

**Published:** 2025-06-03

**Authors:** Lance W.Q. Xu, Steve Pressé

**Affiliations:** 1 Center for Biological Physics, Arizona State University, Tempe, AZ, USA; 2 Department of Physics, Arizona State University, Tempe, AZ, USA; 3 School of Molecular Sciences, Arizona State University, Tempe, AZ, USA

## Abstract

Data modeling tools face trade-offs between accuracy, computational efficiency, data efficiency, and model flexibility. Physics-inspired, rigorous likelihood-based approaches, while offering high accuracy and data efficiency, remain limited in practice due to high computational cost, particularly when applied to larger-scale problems. This general limitation is further compounded by reliance on traditionally single-threaded iterative sampling or optimization procedures, which are difficult to scale. Although prior efforts have attempted to parallelize expensive likelihood-based approaches by partitioning data or running multiple sampling replicas in parallel, such strategies fail for algorithms requiring efficient communication between processes. Here, we introduce a fundamentally different strategy: we exploit the parallelism inherent in both likelihood evaluation and posterior sampling, operating on a single shared dataset. Our framework supports frequent yet lightweight inter-thread and inter-processor communication, making it well-suited for modern parallel architectures. Using diffraction-limited single-particle fluorescence tracking as a case study, this approach achieves up to a 50-fold speedup on a single mid-range GPU compared to conventional single-threaded CPU implementations, demonstrating a scalable and efficient solution for high-performance likelihood-based inference.

## 1 Introduction

**Figure 1:**
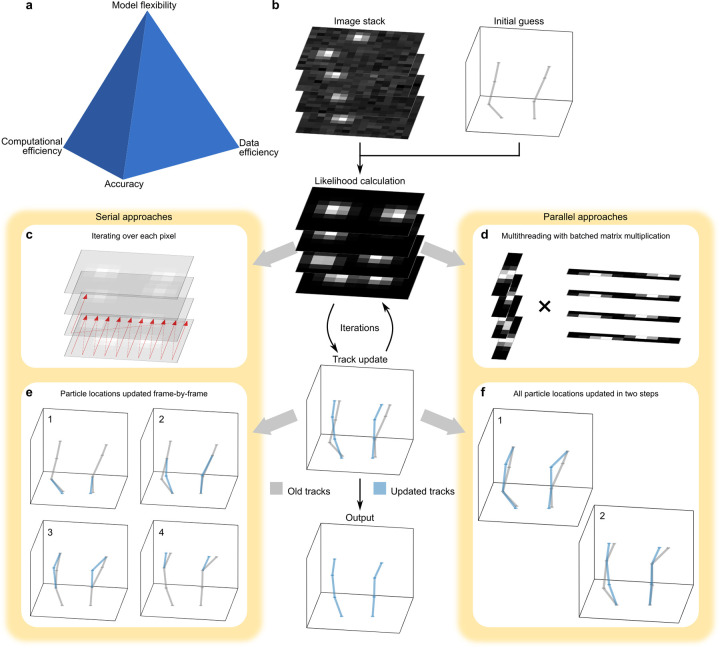
**(a)**, Particle tracking methods often face trade-offs among four key aspects: tracking accuracy, computational efficiency, data efficiency, and model flexibility. **(b)**, In the Bayesian paradigm, tracking begins with a recorded image stack and initial track estimates, followed by iterative updates of particle tracks. These updates rely on evaluating the likelihood of each proposed set of tracks. **(c)**, Likelihood calculations can be performed serially by evaluating each pixel individually. **(d)**, Using batched matrix multiplication allows for parallelized likelihood computation, which can more efficiently leverage thousands of threads available in modern GPUs. **(e)**, A naive approach to updating tracks involves frame-by-frame serial updates. **(f)**, With improved algorithmic design, particle positions at even and odd frames can be updated in just two steps, regardless of how many frames there are in total.

Data modeling tools face key trade-offs between accuracy, computational efficiency, data efficiency, and model flexibility; see Fig. 1a. Among these, physics-inspired likelihood-based approaches represent an essential class due to their foundation in physical principles and mathematical rigor. These methods often yield highly accurate and data-efficient results by directly modeling the generative processes behind observed data^1–17^. However, likelihood-based methods present key limitations. First, constructing a precise likelihood function requires a detailed understanding of the system’s physics, which can restrict the model’s adaptability to different experimental modalities. Second, these methods tend to be computationally intensive, mainly when applied to setups where evaluating the likelihood is computationally demanding and analytically intractable.

To address this computational burden, many methods rely on divide-and-conquer strategies, splitting large datasets into smaller subsets that can be analyzed independently^18–25^. While effective for some tasks, this strategy breaks down when applied to data with strong spatiotemporal correlations, where dividing the data risks discarding essential contextual information. This limitation is particularly relevant across a wide range of scientific disciplines. For instance, particle image velocimetry analyzes flow fields based on tracer particle motion in image sequences^26^, and behavioral studies in neuroscience or ethology rely on continuous video analysis of animal or human subjects^27,28^. In such contexts, accelerating inference without sacrificing information contained in the correlation structure of the data is a key challenge.

Widefield single-particle tracking (SPT) is another representative example of these challenges. Mathematically, a particle’s trajectory is represented as a sequence of spatial positions over time. The objective is to infer the full probability distribution over the number of particle tracks and their associated trajectories, conditioned on the recorded image frames and the physical principles governing the system. This, distribution, termed posterior, is shaped by two key components: the emission model (serving as the likelihood), which quantifies the probability of observing the recorded frames given a set of particle tracks, and the motion model (serving as the prior), which describes the plausibility of the tracks themselves independent of the data.

Accurately evaluating the emission model requires calculating the expected photon emissions from all particles across every pixel and frame, owing to the spreading of light caused by diffraction and the detector’s physical properties. As the number of particles, pixels, and frames increase, this task becomes computationally intensive. In addition, posterior inference typically relies on iterative sampling techniques—most notably, Markov chain Monte Carlo (MCMC)^29^—which, though statistically rigorous, demand repeated evaluations of costly likelihood functions. In our previous work^30^, we demonstrated a physics-inspired SPT framework, BNP-Track, that yields high tracking accuracy and strong data efficiency, particularly under challenging imaging conditions. However, it comes at a significant computational cost: generating sufficient posterior samples often takes several hours on a standard desktop CPU. This contrasts with conventional tools such as TrackMate^31^, which complete inference in seconds. Such computational demands limit the practicality of physics-inspired methods in high-throughput applications, particularly those involving larger biological and imaging datasets.

Approximations are often invoked to help mitigate computational expense. For instance, in the context of particle tracking, a common simplification involves focusing solely on identifying the most probable set of trajectories, often without quantifying uncertainty^31–33^. Another widely adopted approximation relies on greedy algorithm structures decomposing tracking into sequential steps of localization and linking^31–33^. In the localization step, object positions are detected within each frame independently of the other, extracting spatial coordinates and timestamps. The linking step then connects these localized positions across frames to reconstruct full particle trajectories. By reducing a high-dimensional inference problem into a series of lower-dimensional ones, this modular design enables fast computation, often on the order of seconds per dataset. Moreover, it offers considerable flexibility, allowing researchers to tailor the method by mixing and matching localization and linking algorithms to suit various experimental conditions.

While these approximations offer substantial gains in computational speed and model flexibility, they often come at the cost of reduced tracking accuracy^30^. High-precision localization requires a sufficient number of detected photons per frame^34,35^, a condition difficult to satisfy in live-cell imaging. Increasing illumination intensity to boost photon counts risks photodamage^36^, while extending exposure times introduces motion blur, particularly problematic for fast-diffusing biomolecules. As a workaround, datasets are frequently filtered to include only the brightest signals, excluding weaker ones that may still be biologically relevant. Such filtering introduces selection bias and reduces data efficiency by discarding valuable information embedded in lower-intensity signals.

Beyond approximation-based strategies, deep learning approaches for bioimaging^37–40^ as well as particle tracking offer another computationally attractive option^41–45^. Leveraging deep neural networks, these methods offer high model flexibility and, once trained, are computationally efficient. However, their performance is often sensitive to variations in signal-to-noise ratio (SNR)^45^, and they typically require large, carefully curated training datasets—either simulated or experimentally annotated. As a result, their tracking accuracy and data efficiency can be limited, particularly in challenging or previously unseen imaging conditions^30,32^.

In addition to approximation-based strategies, researchers have explored various parallelization techniques to accelerate likelihood-based inference methods^18–23,25,46–50^. Broadly, these approaches fall into two categories: methods that partition the dataset into independent subsets and analyze each in parallel before aggregating the results^18–23,25^, and methods that replicate the full dataset across multiple threads or processes and combine outputs from independent runs^25,46–50^.

While both strategies can accelerate convergence and are often described as embarrassingly parallel, they fall short in settings where frequent inter-process communication is required. This limitation is particularly acute in particle tracking problems, where perpixel likelihoods must be aggregated to compute frame-level likelihoods, which in turn must be combined to evaluate the full system likelihood. Additionally, strong temporal correlations between particle positions across frames introduce statistical dependencies demanding frequent synchronization and global coordination. As a result, communication overhead becomes the dominant bottleneck, limiting the efficiency gains of parallelization for single-chain inference. Furthermore, combining results from parallel runs typically requires *post hoc* approximations, such as weighted averaging of subposterior samples (posteriors of sub-datasets)^20^ or Gaussian process fit on estimated density values^21^, which may compromise the statistical rigor that fully Bayesian methods aim to preserve.

To address these challenges while preserving the full mathematical rigor and tracking accuracy, we present a parallel SPT framework, BNP-Track 2.0. This implementation capitalizes on the intrinsic parallelism of both likelihood evaluation and posterior sampling by leveraging modern computing architectures, including vectorized operations, multi-threading, and GPU acceleration. By supporting frequent yet lightweight communication across threads (see Fig. 1b), the parallel framework achieves a 50-fold speedup on a mid-range GPU (Nvidia GTX 1060, 6 GB) relative to the original serial framework executed on a similarly priced CPU (Intel i7-7700K). Importantly, this acceleration comes with no data efficiency or inference quality loss, making our framework a practical and scalable solution for high-throughput particle tracking in complex biological environments.

## 2 Results

We begin by demonstrating that the new parallel physics-inspired SPT framework retains the same high level of tracking accuracy as its predecessor, particularly in low SNR conditions. At the same time, it delivers a substantial computational boost to rigorous likelihood-based approaches—achieving nearly a 50-fold speedup when run on a mid-range Nvidia GTX 1060 (6 GB) GPU compared to the original serial implementation on an Intel i7-7700K CPU. We then perform a head-to-head comparison of the two frameworks across various parameter regimes, highlighting how their computational performance scales under different conditions. Finally, we show that when the number of emitting particles is fixed (*i.e*., in a parametric setting), the parallel framework achieves an additional 20-fold speedup, bringing the total acceleration to nearly 200-fold on a single GTX 1060 GPU.

### 2.1 Tracking with low SNR data

**Figure 2:**
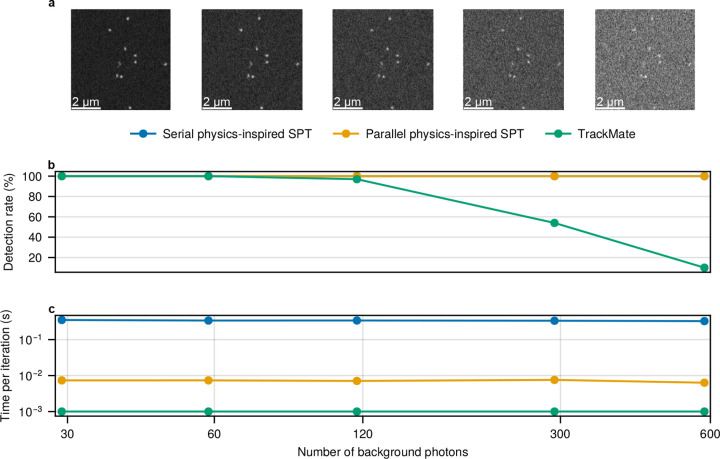
Tracking accuracy and computational efficiency comparisons between the serial physics-inspired SPT, the parallel physics-inspired SPT, and TrackMate^31^ under varying background noise levels for synthetic data. **(a)**, Time-averaged frames corresponding to different background noise levels in terms of the number of background photons per pixel per frame, with contrast adjusted for visualization. Each emitting particle contributes roughly 80 photons to the pixel concentric with the particle’s point spread function (PSF), consistent with Cy3 dye brightness^30,51^. **(b)**, Detection ratio for each method, representing tracking accuracy based on the proportion of true particle detections without spurious results. Note that the detection ratios of the physics-inspired frames (blue and orange) are the same at all noise levels. **(c)**, Per-iteration runtime for each method, representing computational efficiency. The simulated data assumes a numerical aperture of 1.45, a refractive index of 1.515, an emission wavelength of 665 nm, a frame exposure time of 33 ms, and a pixel size of 133 nm, using an EMCCD camera with an offset and EM gain of approximately 100. Each dataset consists of ten 128×128 frames with particles diffusing at 0.1 μm^2^/s. All benchmarks were conducted on the same desktop computer with Intel i7-7700K CPU and Nvidia GTX 1060 6 GB GPU, see Computational efficiency measurement.

In the first robustness test, we verify that the parallel physics-inspired SPT framework preserves its predecessor’s tracking accuracy even for low SNR. We demonstrate this by simulating a series of image stacks sharing the same parameters, including particle tracks, field of view (FOV), pixel size, emission wavelength, numerical aperture, refractive index, and emission rate, and only changing the background level. Each image stack contains ten 128×128 frames and ten diffusing particles. For illustrative purposes, Fig. 2a shows the resulting time-averaged frames; the noise level of the rightmost dataset is 20 times as high as that of the leftmost dataset.

As discussed earlier, many parallel inference methods introduce approximations that can compromise accuracy. It is therefore essential to demonstrate that the proposed parallel physics-inspired SPT framework preserves the accuracy of its serial predecessor. To this end, we compare the tracking performance of the parallel framework, the original serial framework, and TrackMate^31^, a widely used tool in the literature^52–56^. Specifically, we evaluate each method’s accuracy by tuning parameters to ensure that no particle detections occur more than one diffraction limit away from any ground truth position (termed spurious detections). Once this criterion is satisfied, the detection ratio is calculated as the number of correct detections divided by the total number of ground truth positions. As shown in Fig. 2b, both the original and the parallel physics-inspired SPT frameworks reliably detect all particles across all frames. At the same time, TrackMate’s performance degrades significantly as background noise increases.

Having evaluated tracking accuracy using the detection ratio, we now assess computational efficiency. Unlike accuracy metrics, comparing computational efficiency across methods is more nuanced due to inherent differences in what each algorithm infers from the data. For instance, the physics-inspired frameworks estimate particle trajectories and quantify the uncertainty associated with each track—an important capability absent in tools like TrackMate.

Moreover, computational efficiency alone does not fully reflect the practical cost of analysis, particularly for methods requiring extensive manual parameter tuning. TrackMate, for example, asks users to specify parameters such as localization quality thresholds and maximum linking distances. Identifying suitable values often involves a time-consuming, trial-and-error process, the duration of which varies across users and datasets and is challenging to quantify systematically. In contrast, physics-inspired methods generally involve fewer tunable hyperparameters^5,29,30,57,58^, significantly reducing the burden on the user. These methods are typically designed to be fully automated once the input is provided^5,17,57,59–63^, meaning that all time costs are confined to computation itself, with no manual intervention required during execution. As a result, these approaches improve computational efficiency and minimize user effort and variability.

Despite these caveats, comparing per-iteration runtime remains informative, especially in highlighting algorithmic and implementation differences. This comparison is straightforward for physics-inspired SPT frameworks with and without parallelism: both rely on MCMC sampling and follow the same inference logic, requiring a comparable number of iterations to achieve equivalent tracking accuracy. For TrackMate, we define per-iteration runtime as the time taken for one round of localization and linking given a fixed set of parameters—excluding time spent on user tuning—to allow for a fairer comparison of algorithmic performance.

In 2c, we present the per-iteration runtime for each method. Across all signal-to-noise ratio (SNR) regimes, the three methods show similar per-iteration times—an expected result, as SNR primarily affects the number of iterations required for convergence rather than the time per iteration.

TrackMate exhibits the shortest per-iteration time, typically under 10 ms (Intel i7-7700K, 4 cores, 8 threads, up to 4.5 GHz). However, as discussed earlier, this timing excludes the manual effort required for parameter tuning. In contrast, the physics-inspired framework with parallelism, which is algorithmically comparable to its predecessor, achieves over a nearly 50-fold improvement in computational speed on a Nividia 1060 6 GB GPU (see Computational efficiency measurement), underscoring the benefits of the optimizations implemented in the new version.

**Figure 3:**
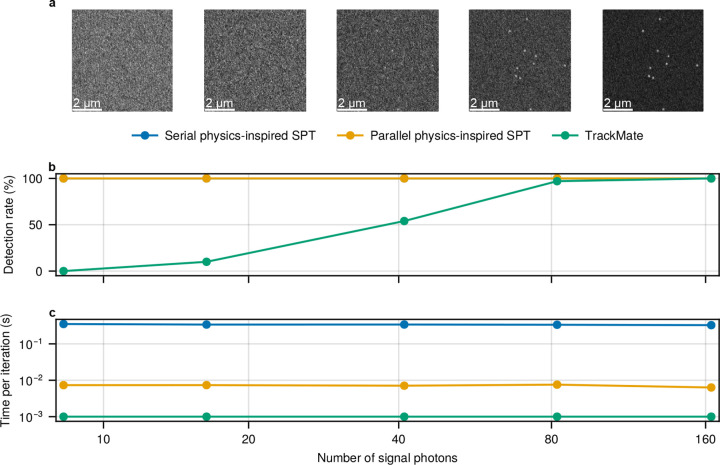
Tracking accuracy and computational efficiency comparisons between the serial physics-inspired SPT, the parallel physics-inspired SPT, and TrackMate^31^ under varying particle emission rates using synthetic data. **(a)**, Time-averaged frames corresponding to different emission rates in terms of the number of photons received at the pixel concentric with the center PSF. The number of 80 photons is consistent with Cy3 dye brightness^30,51^. The amount of background is fixed at around 60 photons per pixel per frame. **(b)**, Detection ratio for each method, representing tracking accuracy based on the proportion of true particle detections without spurious results. Note that the detection ratios of the physics-inspired frames (blue and orange) are the same at all emission rates. **(c)**, Per-iteration runtime for each method, representing computational efficiency. The simulated data assumes a numerical aperture of 1.45, a refractive index of 1.515, an emission wavelength of 665 nm, a frame exposure time of 33 ms, and a pixel size of 133 nm, using an EMCCD camera with an offset and EM gain of approximately 100. Each dataset consists of ten 128×128 frames with particles diffusing at 0.1 μm^2^/s. All benchmarks were conducted on the same desktop computer with Intel i7-7700K CPU and Nvidia GTX 1060 6 GB GPU, see Computational efficiency measurement.

In addition to the tests shown in Fig. 2, we conducted a complementary experiment in which the brightness of each emitting particle varied across datasets. The results, shown in Fig. 3, are consistent with those in Fig. 2, further confirming that the parallel physics-inspired framework maintains its performance while being nearly 50 times more computationally efficient.

### 2.2 Computational efficiency scaling

In the previous section, we demonstrated the computational efficiency gains achieved by the parallel physics-inspired SPT framework. Here, we present a more detailed comparison with its serial counterpart, evaluating how performance scales with frame size, number of frames, and number of emitting particles. In addition to benchmarking the parallel framework on a GPU, we also report its performance on a CPU for a comprehensive assessment across hardware platforms.

**Figure 4:**
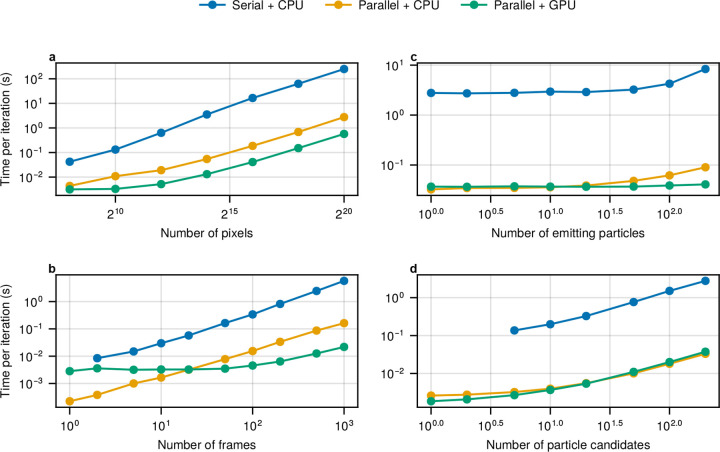
Scalability of computational efficiency for the serial physics-inspired SPT framework on CPU (blue), the parallel framework on CPU (orange), and the parallel framework on GPU (green), across various parameters. **(a)**, Frame size: per-iteration runtime measured as frame dimensions increase from 16×16 to 1024×1024. **(b)**, Number of frames: per-iteration runtime as the number of analyzed frames increases from 1 to 1000. **(c)**, Number of emitting particles: per-iteration runtime as the number of emitting particles increases from 1 to 200 while keeping the frame size and count fixed. **(d)**, Total number of particle candidates: per-iteration runtime when increasing the number of candidates (real and inactive) from 1 to 200 while keeping only one particle active. The simulated data assumes a numerical aperture of 1.45, a refractive index of 1.515, an emission wavelength of 665 nm, a frame exposure time of 33 ms, and a pixel size of 133 nm, using an EMCCD camera with an offset and EM gain of approximately 100. All benchmarks were conducted on the same desktop computer with Intel i7-7700K CPU and Nvidia GTX 1060 6 GB GPU, see Computational efficiency measurement.

In Fig. 4a, we simulate 100 frames, each containing two emitting particles, with frame sizes ranging from 16×16 to 1024×1024 pixels. The parallel physics-inspired SPT framework maintains a tenfold speed advantage on the CPU over its serial counterpart, demonstrating better scaling as frame size increases. This improvement stems from its optimized algorithmic structure, detailed in Methods. On the GPU, the parallel framework achieves an additional fourfold speedup for frame sizes of 32×32 and larger. For 16×16 frames, however, GPU performance is comparable to the CPU due to the relatively small workload per kernel launch and the associated communication overhead between the CPU and GPU^64,65^.

Figure 4b illustrates how the per-iteration time for each method scales with the number of frames analyzed. For this test, we simulated a dataset containing two emitting particles across 1000 frames of size 50×50. Subsets of this dataset were then analyzed, ranging from a single frame to the full 1000-frame stack. Due to its design, the serial framework cannot process datasets with only one frame, rendering it unsuitable for localization tasks. As shown in Fig. 4b and consistent with previous results, the parallel framework achieves approximately a 20-fold speedup on the CPU. However, a computational advantage is only evident for datasets with more than 20 frames using a GPU. For smaller datasets, the communication overhead between CPU and GPU again dominates.

In the following test, we evaluate how the per-iteration time for each method scales with the number of emitting particles. We begin by simulating a dataset of ten 128×128 frames containing a single emitting particle. We then incrementally increase the number of emitting particles up to 200. As shown in Fig. 4c, the per-iteration time of both the serial and the parallel frameworks on the CPU remains relatively stable until the number of emitting particles exceeds 20, beyond which computational cost begins to rise more noticeably. In contrast, the parallel framework running on the GPU remains largely unaffected by the increasing number of emitting particles.

This scaling complexity arises from the use of nonparametric structures, which inherently complicates posterior sampling in physics-inspired frameworks^29,66–68^. In the proposed parallel SPT framework and its serial predecessor, a fixed, user-specified number of candidate particles is assumed, only a subset of which are true emitting particles. The remaining candidates function as “invisible” placeholders. Each candidate is assigned a binary indicator, or load, that determines whether it is active (emitting) or inactive. Since this assignment is updated for all candidates at every iteration, the total number of particles, not just the number of active ones, becomes a key driver of computational cost.

The benefits of combining nonparametric posterior structures with efficient sampling strategies, central to our parallel physics-inspired framework, are further underscored by the results in Fig. 4d, where we fix the number of emitting particles to one while increasing the total number of candidate particles from 1 to 200. As expected, Fig. 4d reveals a more regular scaling behavior consistent with the underlying algorithmic design. Two points are worth noting: first, the original serial framework does not allow the number of candidate particles to be set below five, limiting its flexibility in this regime. Second, with only one emitting particle, the parallel framework exhibits minimal differences in computational efficiency between CPU and GPU implementations. This highlights that GPU parallelism becomes increasingly beneficial only as the number of emitting particles increases.

### 2.3 Nonparametric cost

**Figure 5:**
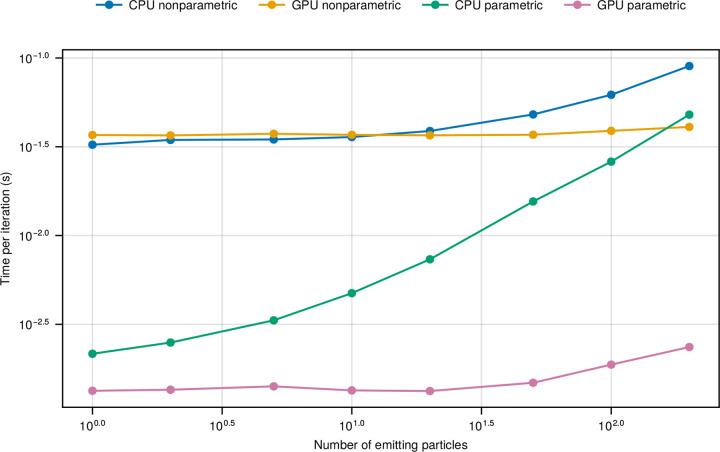
Trade-off between nonparametric flexibility and computational efficiency in our parallel physics-inspired framework for different numbers of emitting particles, illustrated on both CPU and GPU. We compare the performance of the parallel framework in its nonparametric (200 particle candidates, the number of emitting particles ranges from 1 to 200) and parametric (number of candidates equals number of emitting particles) modes, each run on both CPU and GPU. These results highlight a fundamental trade-off: nonparametric formulations offer greater modeling flexibility when the number of particles is unknown or time-varying, but at increased computational cost. The specific needs and constraints of the application should guide the choice between modes. All benchmarks were conducted on the same desktop computer with Intel i7-7700K CPU and Nvidia GTX 1060 6 GB GPU, see Computational efficiency measurement.

Having established the importance of nonparametric structures in physics-inspired frameworks and their role in increasing the complexity of posterior sampling, we now quantify the computational cost associated with this modeling flexibility. To this end, we compare two configurations of the proposed parallel framework: one operating in a nonparametric mode with 200 candidate particles (as in earlier sections), and another in a parametric mode where the number of candidate particles is set equal to the number of true emitters. Both configurations are evaluated on CPU and GPU to assess how hardware affects computational performance under different model assumptions. As shown in Fig. 5, the parametric mode consistently outperforms the nonparametric mode in computational efficiency on both platforms. Notably, for datasets with 200 emitters, the GPU-accelerated parametric version achieves an additional 20-fold speedup over its nonparametric counterpart.

This result highlights an important trade-off: while nonparametric models offer greater flexibility and robustness, especially in settings where the number of particles is unknown, they also come with increased computational cost. The choice between parametric and nonparametric formulations should thus be guided by the specific demands of the application, balancing the need for modeling generality against available computational resources.

## 3 Discussion

Integrating realistic physics into data analysis through complex likelihood functions introduces a dramatic increase in computational cost. While embarrassingly parallel methods have been developed to distribute these computations across large CPU clusters^18–20,22,46–50^, such approaches are most effective when the underlying inference task requires little to no communication between processes. However, this assumption breaks down when modeling systems governed by tightly coupled physical constraints—such as time-correlated motion in particle tracking—where frequent communication between frames or model components is essential. In these scenarios, accelerating the performance of a single inference chain remains a critical bottleneck.

To address this challenge in the context of SPT, we introduce BNP-Track 2.0, a parallel, physics-inspired framework that takes a fundamentally different approach toward computational efficiency. Instead of distributing independent MCMC chains or partitioning datasets across nodes, this parallel framework exploits the fine-grained parallelism embedded within the likelihood structure to speed up a single inference chain. By parallelizing both the likelihood evaluation and associated sampling updates, the framework leverages modern desktop-class hardware with a single GPU to achieve substantial speedups, eliminating the need for large-scale computing infrastructure while retaining the full statistical rigor of Bayesian inference. Importantly, our parallel physics-inspired framework preserves its serial counterpart’s high accuracy and data efficiency, while achieving a nearly 50-fold improvement in computational speed. This substantial performance gain transforms the framework from a powerful but time-intensive tool into one capable of supporting high-throughput analyses. This parallel framework extends the practical reach of likelihood-based inference in analyzing complex spatiotemporal data by bridging the gap between physical rigor and computational efficiency.

As our proposed framework does not rely on embarrassingly parallel strategies, it remains fully compatible with them. Such independence means the framework can be further optimized by incorporating embarrassingly parallel techniques, such as running multiple inference chains or distributing data across nodes, potentially unlocking even greater computational efficiency without compromising its core design. As a result, the framework is well-positioned to scale beyond desktop environments and tackle large-scale data analysis tasks, such as processing the massive volumetric datasets produced by lattice light-sheet microscopy^69^.

Beyond hardware improvements, such as utilizing TPUs for enhanced performance, several emerging computational techniques could further optimize our parallel framework. We highlight three promising directions: (1) Mixed precision computing^70–72^, which allows our framework to treat different variables with different precision levels. For instance, object positions can be stored at lower precision, while probability density estimates of track configurations can be maintained at higher precision. This strategy reduces memory usage and accelerates computation without sacrificing accuracy. (2) Randomized numerical linear algebra^73^, which can speed up matrix operations by leveraging stochastic approximations instead of exact computations, further improving the scalability of our parallel framework in high-dimensional tracking problems. (3) Deep learning-based acceleration^25,74,75^, which can be employed to approximate computational bottlenecks in physics-inspired inference. For instance, deep architectures such as normalizing flows^76^ or diffusion models^77,78^ can be trained to approximate complex likelihoods or to propose efficient samples from challenging posterior distributions^74^.

By offering concrete strategies for principled, non-naive parallelization that preserves mathematical rigor and statistical accuracy, we hope to encourage the adoption of physics-inspired likelihood-based data analysis methods equipped with the parallelism provided by modern computing architectures. Through using techniques such as vectorization, multi-threading, and GPU acceleration, we are no longer limited to either small and simple systems or systems where likelihood can be embarrassingly parallelized^18,19,22,46–50^. Moreover, thanks to the development of modern programming languages and libraries^79–83^, scientists can now implement these acceleration techniques with much lower entry barriers.

We envision a growing role for scalable, interpretable, and parallel likelihood-based frameworks that match the speed of black-box or approximated models and retain transparency and physical grounding. As data grows in volume and complexity, bridging the gap between statistical rigor and computational scalability will be crucial. BNP-Track 2.0 represents one step toward this goal, offering a viable solution for tracking fast, noisy, and crowded biological systems without compromising accuracy or data efficiency.

## 4 Methods

This section introduces the core parallelization and optimization strategies employed in the proposed parallel framework. For clarity, we define the following notation: let N denote the number of frames; I and J represent the number of pixels in the x- and y-directions per frame, respectively; and $w_{n}^{ij}$ denote the binary measurement collected at pixel (i,j) in frame n. Let M be the number of emitting particles, with $x_{n}^{m}$ representing the position of particle m at frame n. The complete dataset can then be written as $w_{1:N}^{1:I,1:J}=\left\{w_{n}^{ij}|n=1,\ldots ,N;,i=1,\ldots ,I;,j=1,\ldots ,J\right\}$, and the full set of particle tracks as $x_{1:N}^{1:M}$.

As discussed in the Introduction, the goal of tracking is to establish a full probability distribution over M (the number of particles) and $x_{1:N}^{1:M}$ (all elements of all tracks), given $w_{1:N}^{1:I,1:J}$ (the recorded frames). In addition, we will also need to estimate the mean squared displacement (MSD) of the particles, as it is typically unknown *a priori*. Using the notations introduced so far, we can express this target probability distribution as $P\left(M,x_{1:N}^{1:M},\;\text{MSD}|w_{1:N}^{1:I,1:J}\right)$.

In the Bayesian paradigm, this target probability distribution is termed the posterior distribution, representing the probability distribution of all variables of interest given the collected data. To construct this posterior, we apply Bayes’ theorem and get

(1) $$P\left(M,x_{1:N}^{1:M},\;\text{MSD}|w_{1:N}^{1:I,1:J}\right)=\frac{P\left(w_{1:N}^{1:I,1:J}|M,x_{1:N}^{1:M},\;\text{MSD}\right)P\left(M,x_{1:N}^{1:M},\;\text{MSD}\right)}{P\left(w_{1:N}^{1:I,1:J}\right)}.$$

On the right hand side, the first term in the numerator, $P\left(w_{1:N}^{1:I,1:J}|M,x_{1:N}^{1:M}\right.,\;\text{MSD})$, represents the likelihood, which is the probability density of the data given the variables of interest. This term usually describes the system’s physics (the model for photon detection in this context) and realistic complications such as hot pixels. The second term, $P\left(M,x_{1:N}^{1:M}\right.,\;\text{MSD})$, known as the prior, imposes additional constraints on the variables of interest, such as a particle’s motion model and ensuring the MSD remains non-negative. The denominator, $P\left(w_{1:N}^{1:I,1:J}\right)$, is referred to as the evidence and is generally treated as a normalization constant, as it does not depend on any variable of interest.

Our parallel framework employs Gibbs sampling^29,84^ to infer the variables of interest. This iterative procedure updates each variable by sampling from its conditional posterior distribution, holding the others fixed. Specifically, the particle trajectories $x_{1:N}^{1:M}$ are first updated from the conditional posterior $P\left(x_{1:N}^{1:M}|M\right.,\;\text{MSD},\left.w_{1:N}^{1:I,1:J}\right)$, followed by updating the number of emitting particles M via $P\left(M|x_{1:N}^{1:M},\;\text{MSD},w_{1:N}^{1:I,1:J}\right)$, and finally, the mean squared displacement MSD is updated using $P\left(\text{MSD}|x_{1:N}^{1:M},M,w_{1:N}^{1:I,1:J}\right)$.

### 4.1 Likelihood parallelization

As introduced above, the likelihood $P\left(w_{1:N}^{1:I,1:J}|M,x_{1:N}^{1:M},\;\text{MSD}\right)$ represents the probability distribution of the data given the number of particles, the particle tracks, and the MSD. Since it describes the system’s physics, it is usually the most computationally expensive term in the posterior distribution. Therefore, we first focus on parallelizing this term to improve the overall computation time of the framework.

To express this likelihood mathematically, we first observe that once all particle tracks are known, neither the number of particles nor the MSD affects photon detection. This observation allows us to simplify $P\left(w_{1:N}^{1:I,1:J}|M,x_{1:N}^{1:M},\;\text{MSD}\right)$ to $P\left(w_{1:N}^{1:I,1:J}|x_{1:N}^{1:M}\right)$.

Furthermore, considering that we are dealing with fluorescently emitting particles whose lifetimes (on the order of nanoseconds^85^) are significantly shorter than the frame period (microseconds and above), the probability that an emitting particle excited during the exposure period of one frame will result in a photon detection in the next frame is negligible. Therefore, it is reasonable to assume that particle positions at frame n do not influence other frames. This assumption allows us to further simplify the likelihood as a product:

(2) $$P\left(w_{1:N}^{1:I,1:J}|x_{1:N}^{1:M}\right)=\prod_{n=1}^{N}P\left(w_{n}^{1:I,1:J}|x_{n}^{1:M}\right).$$

Similarly, since the pixels of a detector usually operate independently, we can express the per-frame likelihood $P\left(w_{n}^{1:I,1:J}|x_{n}^{1:M}\right)$ as a product over the individual pixel likelihoods:

(3) $$P\left(w_{n}^{1:I,1:J}|x_{n}^{1:M}\right)=\prod_{p=1}^{P}P\left(w_{n}^{ij}|x_{n}^{1:M}\right).$$

The per-pixel likelihood $P\left(w_{n}^{ij}|x_{n}^{1:M}\right)$ is influenced by four key factors: the point spread function (PSF) of the optical system, the pixel area $A_{ij}$, the brightness of the particles h, and the number of emitting particles M. The PSF characterizes how light from a point source is distributed across the detector^35^. At the same time, brightness represents the expected number of photons arriving at a pixel perfectly aligned with the PSF of a particle located at the focal plane. The total number of photons received by pixel p from all emitting particles, denoted as $u_{n}^{ij}$, can be expressed as:

(4) $$u_{n}^{ij}=h\sum_{m=1}^{M}\iint_{A_{ij}}\text{PSF}\left(q_{x},q_{y};x_{n}^{m}\right)dq_{x}\;dq_{y}.$$

Then $P\left(w_{n}^{ij}|x_{n}^{1:M}\right)$ can be constructed using equation (4) and $P\left(w_{n}^{ij}|u_{n}^{ij}\right)$, which is specific to the detector and the experimental conditions. For example, if the detector is a single-photon avalanche diode (SPAD) array, $P\left(w_{n}^{ij}|u_{n}^{ij}\right)$ can be modeled as a Bernoulli distribution with a probability of $1-\text{exp}\;\left(-u_{n}^{ij}\right)$. In contrast, if the detector is a charge-coupled device (CCD), $P\left(w_{n}^{ij}|u_{n}^{ij}\right)$ can be modeled as a Gaussian distribution^35^.

In equation (4), the form of the PSF is often approximated as a Gaussian function of^35^:

(5) $$\text{PSF}\left(q_{x},q_{y};x_{n}^{m}\right)\approx C_{n}^{m}\text{exp}\;\left(-\frac{{\left(q_{x}-x_{n}^{m}\right)}^{2}+{\left(q_{y}-y_{n}^{m}\right)}^{2}}{2v_{n}^{m}}\right),$$

where $C_{n}^{m}$ is some normalization constant and $v_{n}^{m}$ is the variance of the Gaussian function in the lateral direction, which is dependent on the emission wavelength, numerical aperture, and a particle’s z-location. With this approximation, equation (4) can be written as a matrix multiplication:

(6) $$u_{n}^{ij}\approx h\sum_{m=1}^{M}u_{n,x}^{i,m}u_{n,y}^{j,m}.$$

Here, $u_{n,x}^{p,m}=\int \sqrt{C_{n}^{m}}\text{exp}\;\left(-\frac{{\left(q_{x}-x_{m}^{m}\right)}^{2}}{2v_{n}^{m}}\right)dq_{x}$ which can be calculated using the error function. It follows that the expected photon count at all pixels on frame n can be expressed as a matrix multiplication:

(7) $$u_{n}^{1:I,1:J}\approx hU_{n,x}U_{n,y}^{T},$$

where $U_{n,x}$ is an I×M matrix with $u_{n,x}^{p,m}$ being its elements. To calculate $u_{1:N}^{1:I,1:J}$, we can perform matrix multiplications for all N frames in parallel. This operation is known as batched matrix multiplication. It is implemented in many numerical and deep-learning libraries^81–83,86–89^. Similarly, as all elements in $u_{1:N}^{1:I,1:J}$ are calculated, each $P\left(w_{n}^{ij}|u_{n}^{ij}\right)$ can be calculated in parallel as well using techniques such as broadcasting^90–92^.

### 4.2 Two-step track update

As described above, particle trajectories are updated by sampling from the conditional posterior distribution $P\left(x_{1:N}^{1:M}|M\right.,\;\text{MSD},\left.w_{1:N}^{1:I,1:J}\right)$ within the Bayesian framework. Based on the concept of Markov boundary^29,93^, the minimal set of random variables containing all useful information to infer another random variable, our parallel framework achieves this through a two-step update scheme designed to maximize parallel efficiency. First, we compute the conditional distribution $P\left(x_{1:2:N}^{1:M}|x_{2:2:N}^{1:M},M,\;\text{MSD},w_{1:N}^{1:I,1:J}\right)$, where 1:2:N and 2:2:N denote all odd and even frame indices, respectively. By holding the particle positions at all even frames fixed, the positions at odd frames become conditionally independent and can thus be updated in parallel. In the second step, we compute the conditional distribution for the even indices, $P\left(x_{2:2:N}^{1:M}|x_{1:2:N}^{1:M},M\right.,\;\text{MSD},\left.w_{1:N}^{1:I,1:J}\right)$, and update all even frame positions in parallel while holding the odd ones fixed. This alternating blockwise update strategy allows all emitting particle tracks to be refreshed in just two passes, each enabling full parallelization across frames.

### 4.3 Memory management

In addition to leveraging parallelism, our framework enhances computational efficiency through careful memory management. A key optimization involves ensuring contiguous memory allocation for the most computationally intensive components of the algorithm^65,94,95^. In particular, the likelihood term, $P\left(w_{1:N}^{1:I,1:J}|x_{1:N}^{1:M}\right)$, is computed using only the M emitting particles. Our parallel framework dynamically reorganizes particle tracks to optimize this step so that the data associated with emitting particles is stored in a contiguous memory block.

Another key memory management strategy employed in our framework is minimizing data copying and avoiding the overhead associated with garbage collection—reclaiming memory no longer in use^94,96^. To achieve this, our framework preallocates memory for intermediate variables—such as $u_{1:N}^{1:I,1:J}$—at the start of execution and reuses this memory throughout the computation.

Similarly, to minimize communication overhead between the CPU and GPU, our framework is designed so that large data transfers occur only once at the beginning of execution. Only lightweight scalar values are exchanged between the CPU and GPU during runtime, significantly reducing latency and maximizing computational throughput.

### 4.4 Computational efficiency measurement

All the tests presented in this study were performed on a desktop computer equipped with an Intel i7-7700K CPU (four cores, eight threads, up to 4.5 GHz), an Nvidia GTX 1060 6 GB GPU, 64 GB of RAM, and Ubuntu 24.04.2 as the operating system. To evaluate the periteration runtime of BNP-Track^30^ and the proposed parallel framework, each method was executed through an initial test run to account for and complete any initialization overhead. Subsequently, the runtime for 1000 iterations was measured, and the reported per-iteration time was computed by dividing the total runtime by 1000.

### 4.5 Software

Our framework is implemented in Julia^79^, a high-level, high-performance dynamic programming language designed for technical computing. The framework leverages several core Julia packages: CUDA.jl^80^ for GPU acceleration on Nvidia hardware, Makie.jl^97^ for high-quality data visualization, Distributions.jl^98^ for probabilistic modeling and working with statistical distributions, and Flux.jl^83,86^ for efficiently implementing batched matrix operations and numerical routines.

## Data Availability

All data supporting the findings of this study are available as benchmark datasets included with the accompanying code repository.
